# Case Report: Recalcitrant oral lichen planus involving bilaterally buccal mucosae treated with a combination of photodynamic and photobiomodulation therapies

**DOI:** 10.12688/f1000research.146733.1

**Published:** 2024-03-01

**Authors:** Juthamast Trakarnboonkij, Suwat Tanya, Wilairat Sarideechaigul, Ajiravudh Subarnbhesaj, Peera Tabbon, Sajee Sattayut

**Affiliations:** 1 Department of Oral and Maxillofacial Surgery, Khon Kaen University, Nai Mueang, Khon Kaen, 40002, Thailand; 2 Lasers in Dentistry Research Group, Khon Kaen University, Nai Mueang, Khon Kaen, 40002, Thailand; 3 Department of Oral Biomedical Science, Khon Kaen University, Nai Mueang, Khon Kaen, 40002, Thailand; 4 Center for Research and Development of Herbal Health Products, Khon Kaen University, Nai Mueang, Khon Kaen, 40002, Thailand

**Keywords:** Oral lichen planus, Burning sensation, Photosensitizer, Low-intensity laser therapy

## Abstract

**Background:**

Managing recalcitrant oral lichen planus (OLP) can be challenging. Laser therapy has been suggested as an alternative to corticosteroids for treatment. Photodynamic therapy (PDT) is a non-invasive technique that enables the removal of lesions without surgery. Photobiomodulation therapy (PBMT) can promote healing and recovery of the lesions.

**Case presentation:**

The objective was to treat unresponsive bilateral OLP of the whole buccal mucosae with a combination of PDT and PBMT.

**Results:**

A 43-year-old Thai male presented with the severe painful reticular type of OLP of bilateral buccal mucosae involving upper and lower vestibular areas. The lesions were not remitted with either prednisolone systemic steroids or fluocinolone topical corticosteroids. After undergoing ten sessions of PDT with 10% 5-Aminolevulinic acid (5-ALA) in the form of thermoplastic gel and a 635 nm diode laser at 100 to 400 mW with an energy density of 20 to 30 J/cm ^2^ in continuous wave mode, combined with five interim-sessions of PBMT using a 635 nm diode laser at 200 to 300 mW with an energy density of 6 to 10 J/cm ^2^ in continuous wave, the patient reported relief of burning sensation beside remission of lesions without any complications.

**Conclusion:**

The wide-spreading recalcitrant OLP with burning sensation can be managed by combining PDT and PBMT.

## Introduction

Oral lichen planus (OLP) is a chronic mucocutaneous inflammatory condition affecting the oral mucosa. ^1^ Among various causes, the cell-mediated immune response is postulated to be a primary factor of this lesion. OLP typically affects the oral cavity bilaterally, including buccal mucosae, tongue, and gingivae. ^2^ It has several types, including reticular, papular, plaque-like, atrophic, erosive, and bullous. ^3^^,^^4^ OLP is a potentially malignant disorder, according to WHO. ^5^^,^^6^ The risk of developing cancer during observational periods ranging from 0.5 to 20 years is between 0.4% and 5%. ^7^^,^^8^ and is higher for smokers, drinkers, and those with hepatitis C. ^8^

Most cases of OLP are incurable lesions. The goal of treating OLP is to reduce lesion size, alleviate symptoms, and improve daily activities. This can decrease the risk of malignant transformation. ^1^ A scoring system ranging from 0 to 5 can be used to evaluate treatment success. ^9^

OLP can be managed by pharmacological and non-pharmacological treatment. Topical corticosteroids are the first-line treatment for frequent recurrences of the condition. However, long-term use or higher doses may be required. Unfortunately, these medications can cause side effects, such as candidiasis, xerostomia, and mucosal atrophy. ^10^^,^^11^ In addition, a study revealed that approximately 20% of patients did not experience any improvement with topical corticosteroid use. ^12^

Laser therapy has been reported as an alternative non-pharmacological treatment for treating recalcitrant OLP. ^11^^,^^13^^–^^15^ Systematic reviews and meta-analysis studies have shown that photodynamic therapy (PDT) and photobiomodulation therapy (PBMT) are effective laser therapy techniques used for OLP without clinical complication and side effect. ^15^^,^^16^

PDT utilizes light to activate a photosensitizer, which alters tissue oxygen forms, reducing hyperproliferation and inflammation. The success of PDT depends on factors such as available tissue oxygen, light source, and type of photosensitizer used. ^11^ Diode lasers with a wavelength from 600 to 800 nm are frequently employed as light sources. ^17^ An effective photosensitizer for treating OLP is 5-ALA, ^18^^–^^21^ but it can be diluted by saliva. It was suggested that formulating 5-ALA in a gel could enhance its effectiveness. ^22^ Currently, the 5-ALA gel is not yet available in any commercial products for use on oral mucosa.

PBMT uses a low-intensity laser to stimulate natural biological processes like cell metabolism, tissue regeneration, and healing. This therapy has a biphasic mechanism. An optimal low-intensity laser dosage enhances biostimulatory effects, while high dosages inhibit cellular activities. This leads to analgesic, anti-inflammatory, and immune modulatory effects. PBMT is a non-invasive and safe treatment without any clinical complications. Studies suggest that PBMT is an alternative treatment for OLP as it can effectively reduce pain and inflammation. ^16^^,^^23^

This case report describes the use of PDT and PBMT to treat oral lichen planus with burning sensation. The report also includes information on the properties and preparation of 5-ALA gel.

## Case report

This case report was approved by the Khon Kaen University Ethics Committee for Human Research, with reference number HE662013. The written informed consent in Thai language for publication of his clinical details and images was obtain from the patient. This consent was also submitted to the ethics committee.

### Patient information and demographic information

A 43-year-old Thai man complained of a severe burning sensation in his bilateral buccal mucosae for one month. The patient experienced worsened symptoms when consuming hot or spicy foods. Despite receiving treatment with 0.1% Fluocinolone acetonide four times a day and taking 10 mg prednisone twice daily, there was no noticeable improvement in the lesion size or symptoms. Therefore, he was referred to the Laser Clinic at the Department of Oral and Maxillofacial Surgery, Faculty of Dentistry, Khon Kaen University.

### Medical history

The patient had no systemic disease based on physical examination and laboratory investigation. He was categorized into ASA PS I.

### Family and psychosocial history

There was no history of medical and psychological disorders of the patient and his family.

### Extraoral and intraoral examination

**Figure 1.**
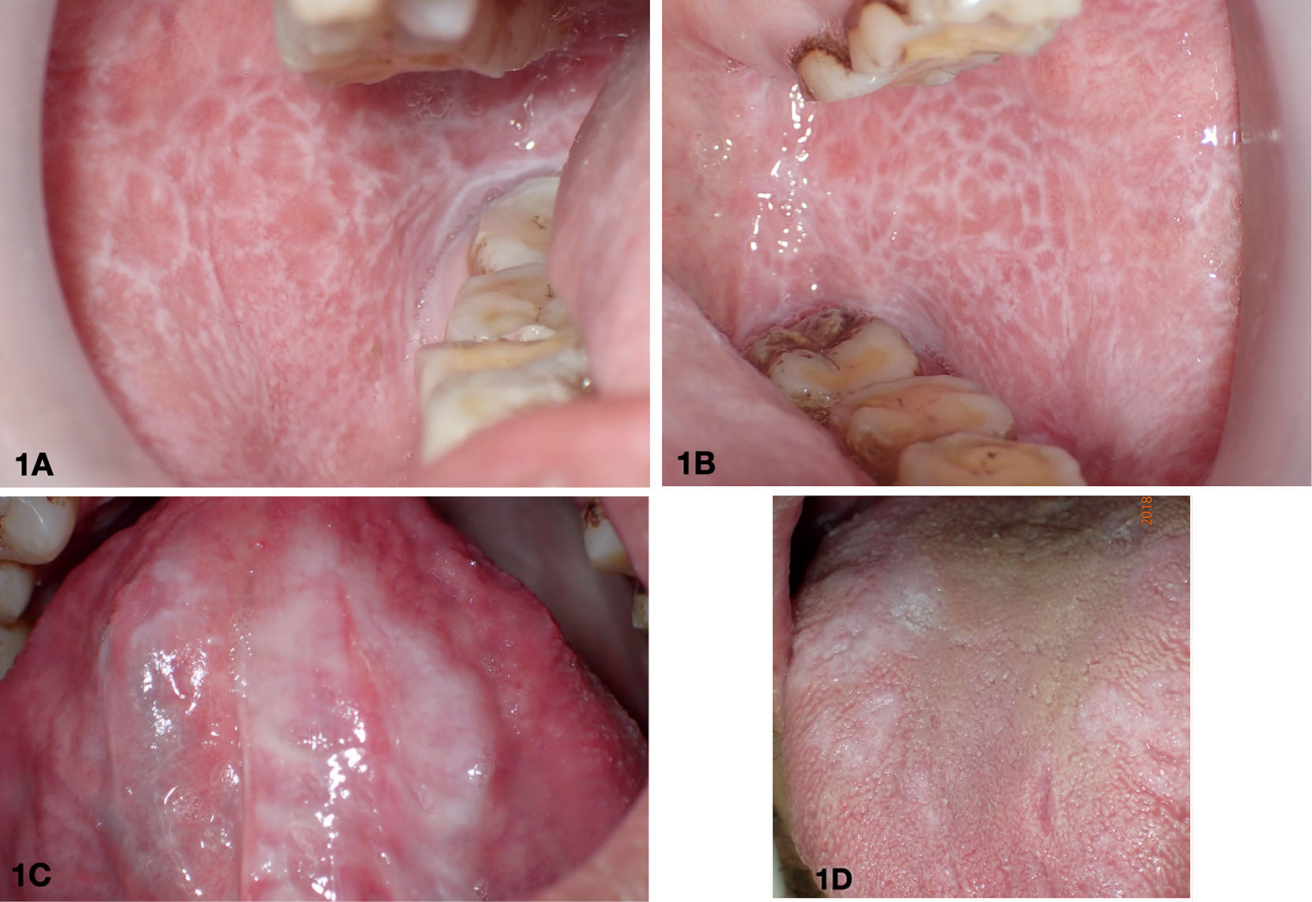
The lesions were observed on the intraoral mucosae: right buccal mucosa (1A), left buccal mucosa (1B), ventral tongue (1C), and dorsal tongue (1D).

From extraoral examination, no significant skin lesions were identified on the patient. The intraoral examination revealed several lesions of the oral mucosae which are shown in Figure 1. The patient had white striae networks in the upper and lower vestibule and retromolar regions on both sides of the buccal mucosae. Additionally, white patches were also present on both the dorsal and ventral surfaces of the tongue.

### Diagnostic evaluation

**Figure 2.**
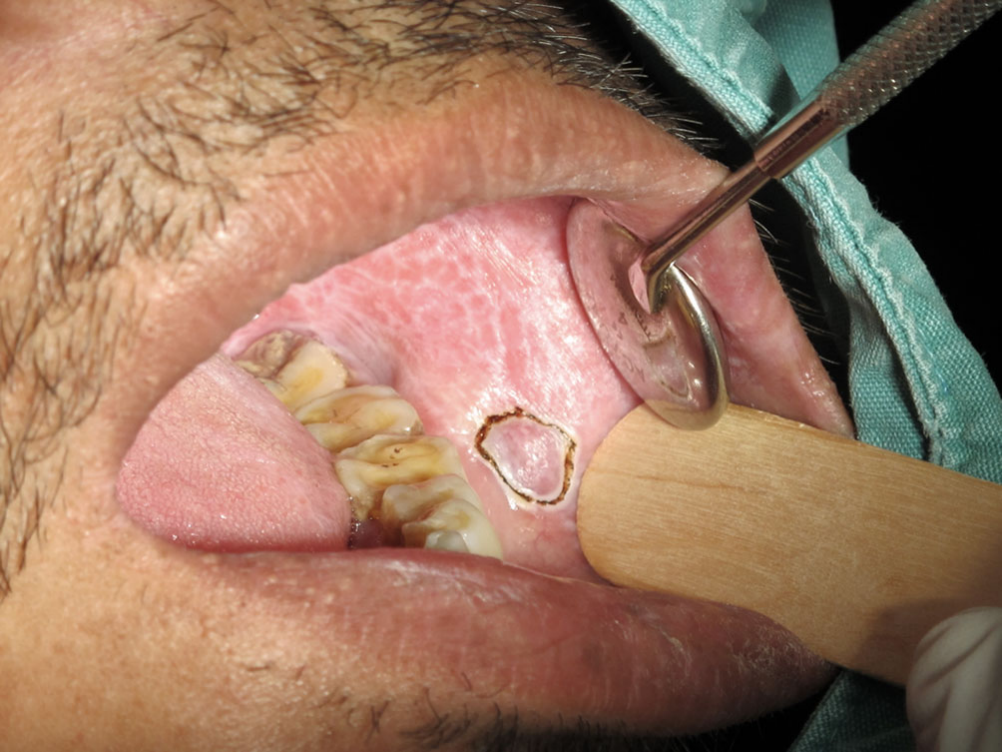
The outline of representative area for incisional biopsy performed by using CO _2_ laser.

**Figure 3.**
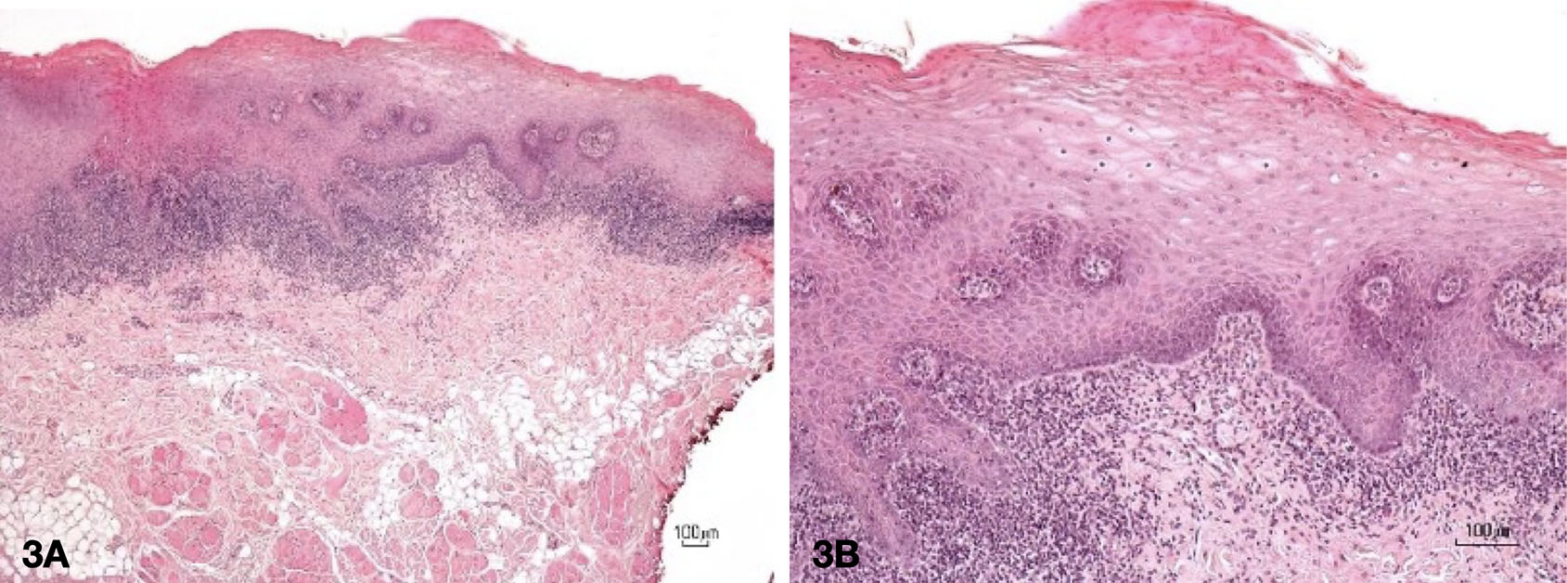
Histopathological investigation showed a distinctively band-like pattern of dense infiltration of lymphohistiocytic cells. The carbonized zone was so narrow at less than 40 microns. This did not interfere with the oral pathologist's observation of the histological sample. (3A magnification at 40x, 3B magnification at 100x).

**Figure 4.**
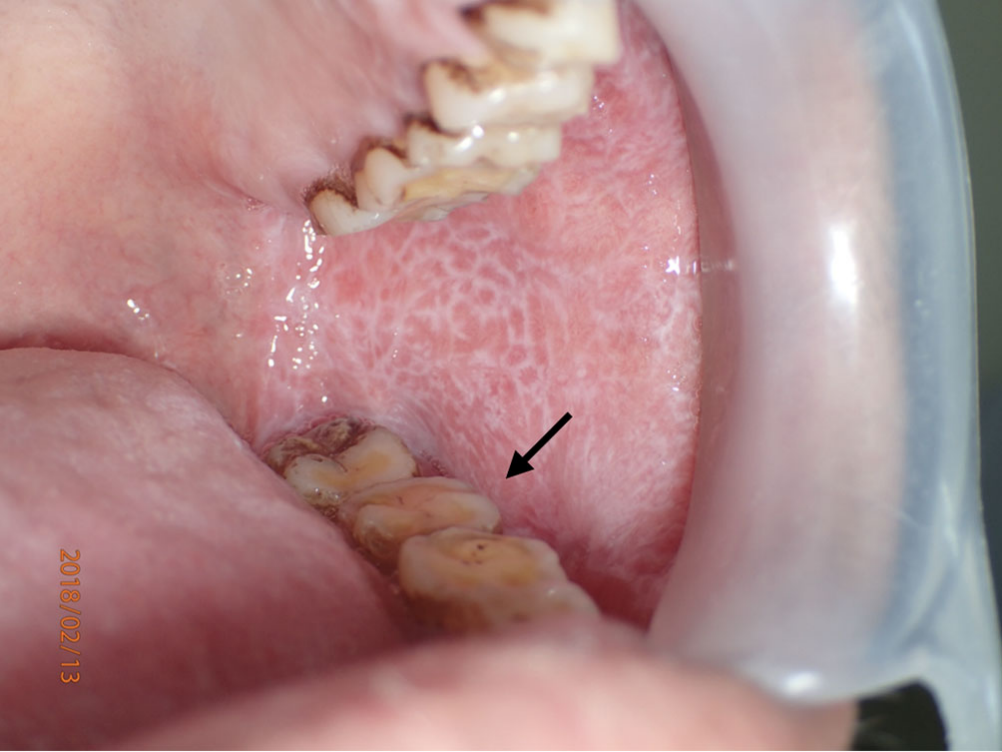
Three weeks after the biopsy, it was found that the tissue had a recurring lesion (arrow).

An incisional biopsy was performed on the left buccal mucosa using a CO _2_ laser at 4W with the continuous wave mode to obtain a specimen. The representative area is shown in Figure 2. Upon histopathological investigation ( Figure 3), a piece of soft tissue covered by parakeratotic acanthotic stratified squamous epithelial with some foci of liquefactive degeneration of the basal cell and apoptotic keratinocytes was observed. The epithelial layer was densely infiltrated by lymphohistiocytic cells in a band-like pattern. These histological features were interpreted as oral lichen planus. No evidence of dysplasia or malignancy was observed in this section. The definitive diagnosis was oral lichen planus. Three weeks later, the affected area showed signs of a recurrent lesion, and the mucosal coverage was complete ( Figure 4).

### Therapeutic interventions

**Figure 5.**
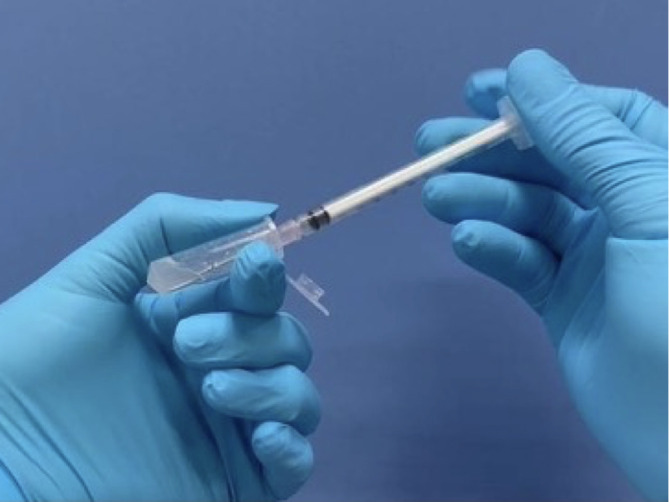
Fresh preparation of photosensitizing gel.

**Figure 6.**
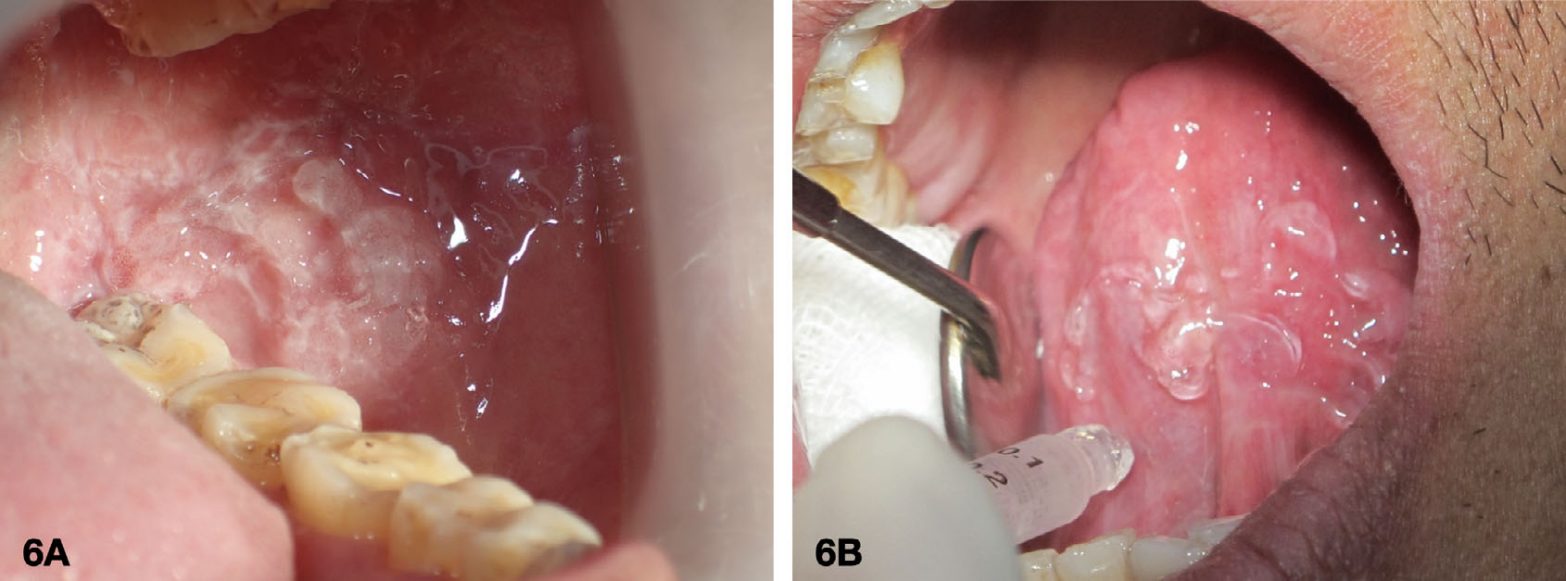
Using a thermoplastic gel base makes it easy to retain 10% 5-ALA photosensitizing gel on the mucosal surfaces, as shown in the pictures of the left buccal mucosa (6A) and the ventral tongue (6B).

**Figure 7.**
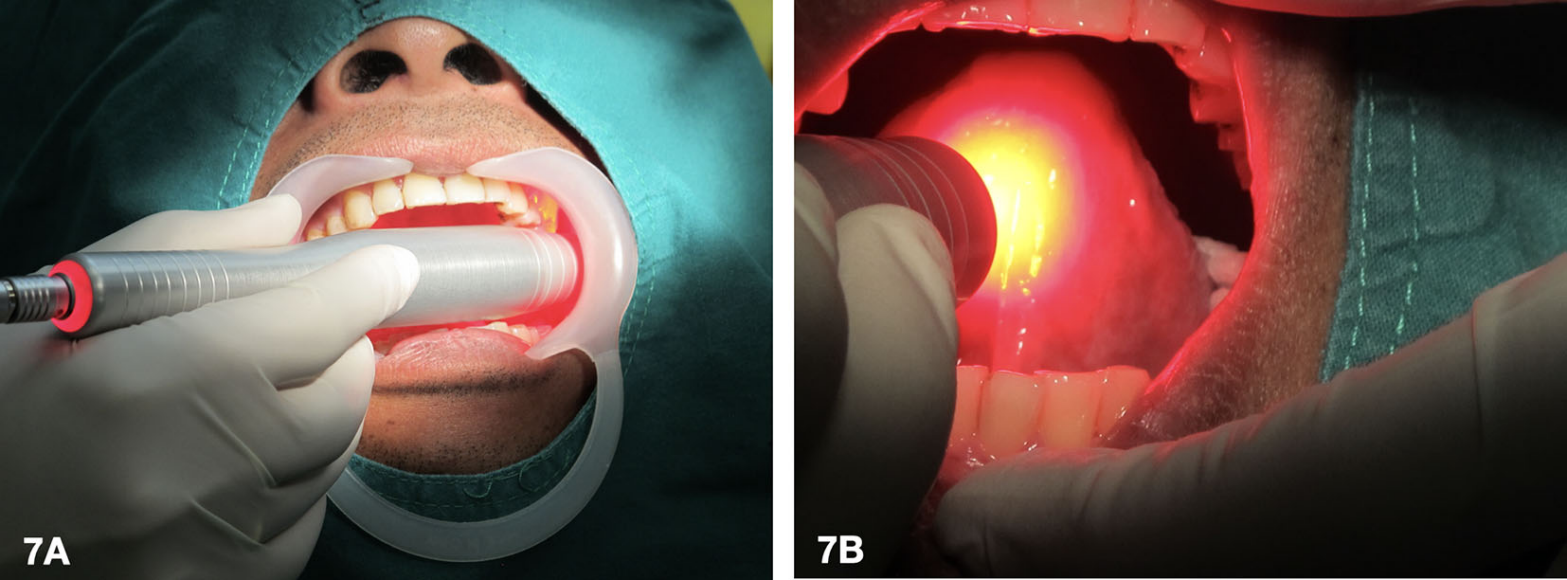
Laser irradiation activates a photosensitizing agent in the tissue of the left buccal mucosa (7A) and the ventral tongue (7B).

• Photodynamic therapy
  1. Preparation and physical properties of gel phase
    The thermoplastic gel used in our PDT underwent gelation through temperature increase. The in-situ gel was prepared by adding 15% (w/w) poloxamer 407 and 0.1% (w/w) sodium benzoate to the mixture on a weight basis (Center for Research and Development of Herbal Health Products, Faculty of Pharmaceutical Sciences, Khon Kaen University, Thailand). Firstly, sodium benzoate was dissolved in cold water, and then poloxamer 407 was added to the solution. The mixture was stirred until it was completely dissolved. Finally, the gel was sterilized using an autoclave (Hariyama HICLAVE HV-85II, Japan).
  2. Physical properties of photosensitizing-containing gel
    To demonstrate the process of gel formation due to thermal increase, we determined the gelation temperature by visually inspecting the sample. We transferred the gel to a test tube and placed it in a water bath with the temperature initially set at 20°C, which 1°C increased until it reached 40°C. We observed the samples visually to see when a gel was formed.
    In vitro, release was investigated using a modified Franz diffusion cell with the non-porous membrane (Cellu Sep, T4). The receiver chamber, equipped with a magnetic stirrer, was filled with Phosphate buffer saline as the receiver medium, and stirred at 300 rpm. The temperature was set at 37±0.5°C. We placed 500 mg of gel in the donor compartment chamber and covered it tightly with paraffin film.
    We collected 0.5 mL of the receiver medium at 0, 15, 30, 60, 90, 120, and 180 min and refilled with 0.5 mL of the warmed fresh medium. The 5-ALA in the medium was converted into a fluorescent derivative with fluorescamine and analyzed by high-performance liquid chromatography with fluorescence detector. ^24^ This method confirmed the favorable properties of the 5-ALA gel preparation, as shown in Table 1.
  3. The preparation of photosensitizing agent ( Figure 5)
    To prepare a fresh batch of gel for 5-ALA, the 5-ALA powder from a vial was mixed with a fluid gel base. The gel base was stored in a 1-ml syringe at 20°C. The resulting mixture was then drawn back into the syringe while still fluid and left at room temperature to solidify. The 5-ALA gel with photosensitizing properties was now ready to use.
  4. Application of a photosensitizing agent on the intraoral lesion
    The sterile gauze was used to clean and dry the mucosa. A fresh gel with 10% of 5-ALA was then applied to the affected area as shown in Figure 6 and left for 30 minutes.
  5. Laser activation of the photosensitizing agent ( Figure 7)
    A 635 nm diode laser was used as a light source to activate the tissue that was applied with the 5-ALA gel. During laser irradiation at 400 mW, the patient experienced a burning sensation. As a result, the laser settings for the first and second sessions were reduced to 100 mW with an energy density of 20 J/cm ^2^ in continuous wave mode. These sessions were conducted every 2 weeks. As the patient could tolerate, the laser dose was increased to 400 mW with an energy density of 30 J/cm ^2^ in continuous wave mode every 3 weeks. In total, there were 5 episodes.

**Table 1.** The results of gel temperature and 5-ALA releasing of the gel compared with the 10% 5-ALA Gel.

Sample	Gelation temperature (°C)	pH	%release (30min)
Gel	32.0±0.0	5.1	-
10% 5-ALA Gel	26.3±0.6	2.2	10.05±1.80%

• PBMT as an interim therapy
  After completing 5 sessions of PDT, the patient underwent PBMT as an interim therapy to aid in the healing process of the affected areas. The therapy involved using a 635 nm diode laser with a power output of 200 to 300 mW and an energy density of 6 to 10 J/cm ^2^ in continuous wave mode. The power and energy density were adjusted incrementally as the patient experienced no discomfort during treatment. A total of 5 sessions were conducted every 2 weeks.
• PDT after PBMT
  The patient underwent PDT for an additional 5 sessions by using 10% 5-ALA gel fresh preparation irradiated with a 635 nm at 400 mW with an energy density of 30 J/cm ^2^ in continuous wave mode. The patient received the treatment once every 3 or 4 weeks.

### Follow-up visits

**Figure 8.**
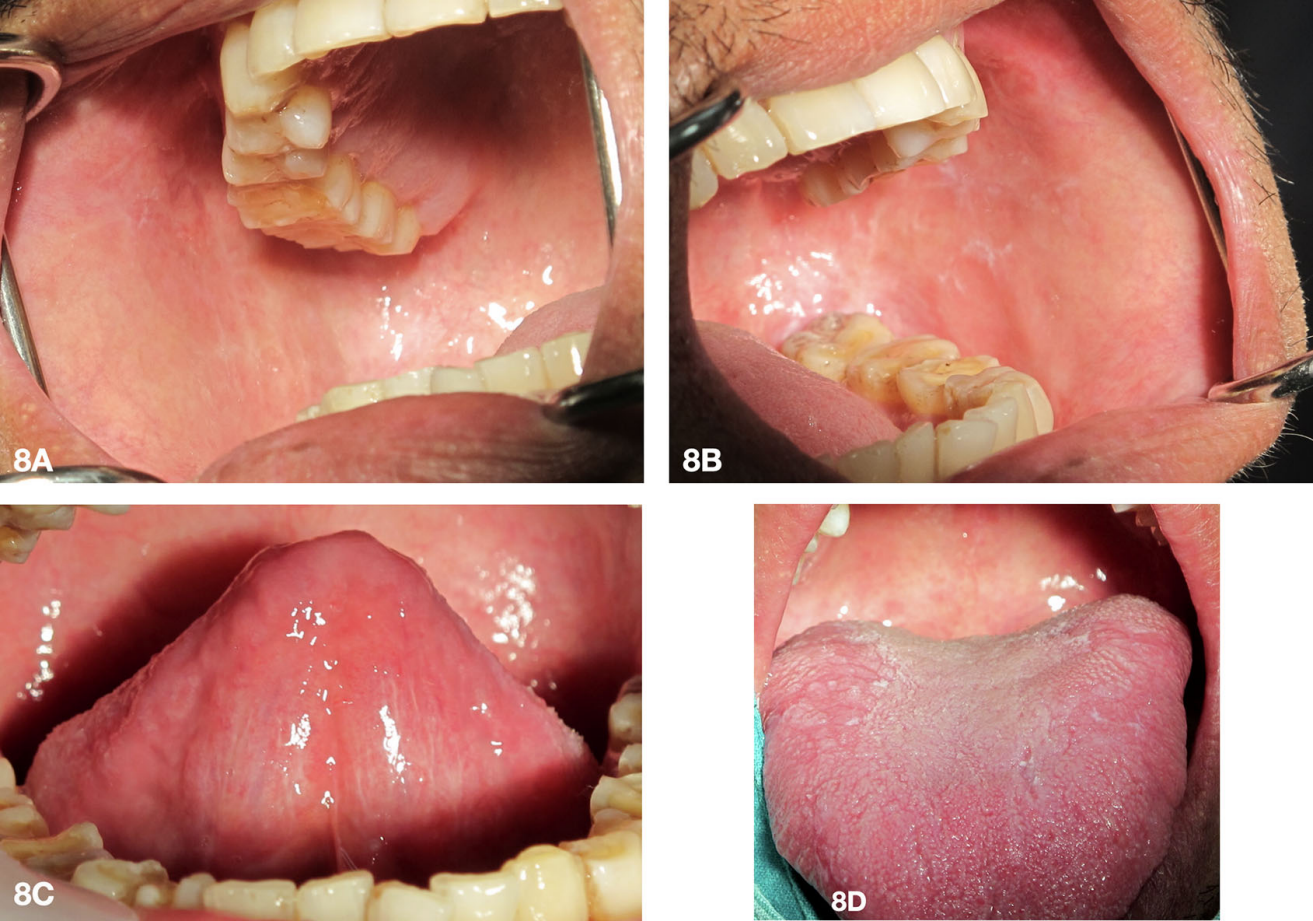
After the patient completed ten sessions of PDT and five interim sessions of PBMT, no significant OLP lesions were observed during the 12-month follow-up. The affected areas, which are the right buccal mucosa (8A), the left buccal mucosa (8B), the ventral tongue (8C), and the dorsal tongue (8D), were examined and showed no signs of significant lesions.

During the initial 5 sessions of PDT and subsequent 5 sessions of PBMT, the patient visited for follow-up appointments every two weeks. The patient reported a decrease in the burning sensation. The lesion size had reduced with a smoother surface of the oral mucosa after the third session of PDT. Gradually, over time, the clinical signs and symptoms improved. Upon completion of 10 PDT sessions, including 5 PBMT sessions in between, the patient successfully reduced lesion size and burning sensation without any clinical complications within a 12-month, as illustrated in Figure 8.

After completing the treatment, the patient consistently attended follow-up appointments every 1 to 2 months for the 3-year follow-up period. During these visits, the patient reported no discomfort or symptoms while consuming spicy food. Additionally, the patient expressed their willingness to return for further evaluation if any symptoms arose.

## Discussion

The primary difficulty in this case was the patient experiencing a burning sensation caused by reticular type OLP, which affected widespread areas of the oral mucosae. Despite using both topical and systemic steroids, there was no significant improvement in the lesions. Due to the size of the oral lesion, it was not possible to remove it surgically with high-intensity laser therapy. Moreover, the previous lesion recurrence after CO _2_ laser biopsy showed that surgery would not be an effective treatment in this particular case. Based on the reasons previously stated, non-pharmacological treatments such as PDT and PBMT seem to be the only viable options for treating this unresponsive case of OLP. These laser therapies are non-invasive and have been proven to treat OLP effectively. ^16^^,^^23^

After undergoing 10 sessions of PDT combined with 5 sessions of interim PBMT, the patient showed partial remission of the lesion. The patient reported a decrease in pain score over a 12-month period. These results align with previous studies using PDT to treat OLP. In fact, a systematic review conducted by Nagi et al. in 2023 found that PDT was an effective treatment option for managing symptomatic OLP that was unresponsive to corticosteroid therapy. This review analyzed ten clinical studies and found that PDT effectively reduced lesion size and VAS scores both during follow-up sessions and after treatment. Furthermore, four of the studies noted that PDT was more effective than topical corticosteroids in reducing the signs and symptoms of OLP, with a *p*-value < 0.01. ^25^ Another systematic review by Al-Maweri et al. also found that PDT was a successful treatment option for patients with symptomatic OLP. ^11^

A study conducted by Sulewska et al. on 50 patients with 124 lesions showed that PDT using 5% 5-ALA and 630 nm lights was effective in treating reticular type OLP. PDT was administered every two weeks for a total of 10 weeks, which resulted in a 62.91% reduction in the size of the lesions. ^19^ Our study administered PDT biweekly, using 10% 5-ALA gel, over a 12-month monitoring period. The higher photosensitizer concentration led to a further reduction in lesion size and decreased pain intensity. Using thermoplastic gel, we also developed an easy method for applying 5-ALA on oral mucosa. The gel form of 5-ALA was partially resistant to saliva dilution and exhibited adhesive properties, facilitating its absorption from the mucosal surface. This was supported by Chen et al.’s study using a 20% 5-ALA sol-gel concentration to treat oral hyperplasia and leukoplakia. ^22^

Another factor of the effectiveness of PDT is highly dependent on the laser parameters used. A systematic review conducted by Al-Maweri et al. reported a lack of consistency in laser parameters used in the included studies. There was a wide range of variation observed in power density (10 to 500 mW/cm ^2^), energy density (1.5 to 15.6 J/cm ^2^), duration of laser irradiation (2 to 10 minutes), and the number of sessions (1 to 8 sessions). Despite these variations, PDT is an effective treatment for symptomatic OLP. ^11^ In this case study, we employed a 635 nm diode laser in continuous wave mode, with a power output that varied between 100 and 400 mW, and an energy density that ranged from 20 to 30 J/cm ^2^. We reduced the dose’s power and energy density to prevent the patient from experiencing a burning sensation during irradiation. In this case, we aimed to induce apoptosis in the hyperkeratotic areas of the lesions while treating symptomatic OLP and promoting mucosal healing. To achieve this, we combined PBMT with PDT. PBMT has a significant impact on biological processes, both biostimulatory and inhibitory. Specifically, it has analgesic effects by increasing the production of ß-endorphins and enkephalins, decreasing the levels of bradykinin and histamine, and modulating pain signalling pathways through C-fiber. Additionally, PBMT can enhance the release of leukocytes into oral tissues and modulate mast cell function, which contributes to inflammation control in the oral mucosa. ^23^

## Conclusions

Using a 635 nm diode laser with 10% 5-ALA-mediated PDT and PBMT was an alternative treatment for recalcitrant OLP. The thermoplastic gel utilized as a base for the photosensitizing agent was suitable for intraoral application.

## Data Availability

No data are associated with this article. Figshare: CARE flowchart and Checklist for Case report: Recalcitrant Oral Lichen Planus Involving Bilaterally Buccal Mucosae Treated with A Combination of Photodynamic and Photobiomodulation Therapies, https://doi.org/10.6084/m9.figshare.24925383.v1. ^26^
