# Supplementary material for: Case Report: Recalcitrant oral lichen planus involving bilaterally buccal mucosae treated with a combination of photodynamic and photobiomodulation therapies
Source: F1000Res. 2024 Mar 1;13:152. [Version 1] doi: 10.12688/f1000research.146733.1 (PMC11162524; doi:10.12688/f1000research.146733.1)
Supplement: Peer review history

# Reviewer report: Reviewer response for version 1

**DOI:** 10.5256/f1000research.160849.r275899

**Published:** 2024-06-08

**Authors:** Alberto Rodriguez-Archilla

**Affiliations:** 1 Universidad de Granada, Granada, Spain

This is an interesting case report of a recalcitrant oral lichen planus treated with a combination of photodynamic with photobiomodulation therapies. The manuscript contains four keywords, eight figures with twelve sections, one table, and twenty-six references. Overall, it is a correct, complete, and well-documented case report.

 Study Strengths

 - The authors report a case of a 43-year-old male patient with oral lichen planus (OLP) whose lesions did not resolve with either prednisolone systemic steroids (prednisolone) or topical corticosteroids (fluocinolone). After undergoing ten sessions of photodynamic therapy (PDT) with 10% 5-Aminolevulinic acid (5-ALA) in the form of thermoplastic gel and a 635 nm diode laser at 100 to 400 mW with an energy density of 20 to 30 J/cm2 in continuous wave mode, combined with five interim-sessions of photobiomodulation therapy (PBMT) using a 635 nm diode laser at 200 to 300 mW with an energy density of 6 to 10 J/cm2 in continuous wave, the patient reported relief of burning sensation beside remission of lesions without any complications.

 - As this paper highlights, some oral lichen planus (OLP) lesions do not respond adequately to corticosteroid therapy. Photodynamic and photobiomodulation therapies could be an alternative therapeutic option for the treatment of recalcitrant OLP lesions. Photodynamic therapy (PDT) favors the removal of lesions without surgery. Photobiomodulation therapy (PBMT) can promote healing and recovery of the OLP lesions.

 Study Weaknesses

 - Case reports include some limitations such as they can have the bias to publish particularly favourable results;  they have no epidemiological value because it is not possible to infer causality; they are necessarily retrospective, so they may lack some important uncollected data.

 - A single case report is not sufficient to adequately assess the efficacy of therapeutic alternatives (PDT, PBMT) in the treatment of oral lichen planus.

 Some additional remarks are made on different sections of the manuscript.

 Keywords

 The manuscript presents four keywords. For keywords, where possible, please use Medical Subject Headings terms (MeSH Terms). Strictly, none of them is a MeSH term. Some alternative MeSH terms proposed could be “lichen planus, oral” better than “oral lichen planus”; "photosensitizing agents" rather than “photosensitizer”; "low level light therapy" or

 "laser therapy" instead of “low-intensity laser therapy”. Nevertheless, these suggestions about keywords are optional, not mandatory.

 Other manuscript sections

 Abbreviations and acronyms, even well-known ones, should be explained the first time they are used, e.g. 5-ALA, ASA PS I, etc.

 To make text understanding easier, if the author's name appears in the text, place the reference number immediately after the name, not at the end of the sentence or paragraph. Please, revise this, especially in the discussion section.

 References

 Total number of the manuscript references: 26.

 Figures

 Total number of the manuscript figures: 8 (figure 1 A, B, C, and D; figure 2; figure 3 A and B; figure 4; figure 5; figure 6 A and B; figure 7 A and B; figure 8 A, B, C, and D).

 The figures have appropriate figure legends.

 Tables

 Total number of the manuscript tables: 1.

 The table has an appropriate title and information. Nevertheless, the abbreviation 5-ALA should be explained.

Are enough details provided of any physical examination and diagnostic tests, treatment given and outcomes?

Yes

Is the case presented with sufficient detail to be useful for other practitioners?

Yes

Is sufficient discussion included of the importance of the findings and their relevance to future understanding of disease processes, diagnosis or treatment?

Yes

Is the background of the case’s history and progression described in sufficient detail?

Yes

Reviewer Expertise:

Oral pathology and medicine.

I confirm that I have read this submission and believe that I have an appropriate level of expertise to confirm that it is of an acceptable scientific standard.

# Reviewer report: Reviewer response for version 1

**DOI:** 10.5256/f1000research.160849.r264352

**Published:** 2024-04-26

**Authors:** Chun Hung Chu

**Affiliations:** 1 The University of Hong Kong, Hong Kong, Hong Kong

This manuscript is well-organised and provides valuable laser application parameters for treating oral lichen planus (OLP). It offers significant guidance for clinical laser applications targeting OLP. 

 Here are some minor suggestions: 

 1. In the abstract, the case presentation section could include the patient's main complaint, the progression of the disease, and the clinical examination. For example, it would be helpful to mention the duration of the disease, previous therapies attempted (to correspond with the title "recalcitrant"), and the size of the oral lesion. By incorporating this information, the beginning part of the results section could be moved to the case presentation section. 

 2. For figures 1 and 8, it would be beneficial if the authors adjusted the pictures to the same size, as it would improve the overall appearance and tidiness. 

 3. As a follow-up case report, it would be more compelling if the authors could provide contrasting intraoral photos after the initial 5 sessions of photodynamic therapies and subsequent 5 sessions of photobiomodulation therapies, as well as photos taken after a 3-year follow-up. This additional evidence would strengthen the article. 

 Overall, this manuscript is meaningful and provides valuable insights.

Are enough details provided of any physical examination and diagnostic tests, treatment given and outcomes?

Yes

Is the case presented with sufficient detail to be useful for other practitioners?

Yes

Is sufficient discussion included of the importance of the findings and their relevance to future understanding of disease processes, diagnosis or treatment?

Yes

Is the background of the case’s history and progression described in sufficient detail?

Yes

Reviewer Expertise:

Preventive Dentistry, Community Dentist, Fluoride Research, Dental LASER

I confirm that I have read this submission and believe that I have an appropriate level of expertise to confirm that it is of an acceptable scientific standard.
